# Somatosensory Profiles but Not Numbers of Somatosensory Abnormalities of Neuropathic Pain Patients Correspond with Neuropathic Pain Grading

**DOI:** 10.1371/journal.pone.0043526

**Published:** 2012-08-22

**Authors:** Karl-Heinz Konopka, Marten Harbers, Andrea Houghton, Rudie Kortekaas, Andre van Vliet, Wia Timmerman, Johan A. den Boer, Michel M. R. F. Struys, Marten van Wijhe

**Affiliations:** 1 Department of Anesthesiology, Pain Management Unit, University Medical Center Groningen, University of Groningen, Groningen, The Netherlands; 2 Department of Psychiatry, University Medical Center Groningen, University of Groningen, Groningen, The Netherlands; 3 Merck & Co., Inc., West Point, Pennsylvania, United States of America; 4 Department of Neuroscience, Neuroimaging Center, University Medical Center Groningen, University of Groningen, Groningen, The Netherlands; 5 PRA International, Zuidlaren, The Netherlands; Hospital Nacional de Parapléjicos, Spain

## Abstract

Due to the lack of a specific diagnostic tool for neuropathic pain, a grading system to categorize pain as ‘definite’, ‘probable’, ‘possible’ and ‘unlikely’ neuropathic was proposed. Somatosensory abnormalities are common in neuropathic pain and it has been suggested that a greater number of abnormalities would be present in patients with ‘probable’ and ‘definite’ grades. To test this hypothesis, we investigated the presence of somatosensory abnormalities by means of Quantitative Sensory Testing (QST) in patients with a clinical diagnosis of neuropathic pain and correlated the number of sensory abnormalities and sensory profiles to the different grades. Of patients who were clinically diagnosed with neuropathic pain, only 60% were graded as ‘definite’ or ‘probable’, while 40% were graded as ‘possible’ or ‘unlikely’ neuropathic pain. Apparently, there is a mismatch between a clinical neuropathic pain diagnosis and neuropathic pain grading. Contrary to the expectation, patients with ‘probable’ and ‘definite’ grades did not have a greater number of abnormalities. Instead, similar numbers of somatosensory abnormalities were identified for each grade. The profiles of sensory signs in ‘definite’ and ‘probable’ neuropathic pain were not significantly different, but different from the ‘unlikely’ grade. This latter difference could be attributed to differences in the prevalence of patients with a mixture of sensory gain and loss and with sensory loss only. The grading system allows a separation of neuropathic and non-neuropathic pain based on profiles but not on the total number of sensory abnormalities. Our findings indicate that patient selection based on grading of neuropathic pain may provide advantages in selecting homogenous groups for clinical research.

## Introduction

The International Association for the Study of Pain (IASP) defined neuropathic pain as a direct consequence of a lesion or disease affecting the somatosensory system [1]. Neuropathic pain has traditionally been classified based on the underlying aetiology [2]; [3]; [4]. Due to the lack of a specific diagnostic tool for neuropathic pain, a grading system of ‘definite’, ‘probable’, ‘possible’ and ‘unlikely’ neuropathic pain was proposed [1]. This grading system aims to determine with a greater level of certainty whether a pain condition is neuropathic, especially relevant when including patients in clinical trials. Briefly, the grade ‘unlikely’ is applicable when patients lack a history of a lesion or disease with a plausible neuroanatomical distribution of their pains. The grade ‘possible’ could be regarded as a working hypothesis, which does not exclude, neither diagnoses neuropathic pain. Patients who fall into the category ‘possible’ neuropathic pain can be transferred into the grades ‘probable’ and ‘definite’ if neurologic examination and the presence of a positive confirmatory test reveal confirmatory evidence. Only the grades ‘probable’ and ‘definite’ indicate neuropathic pain.

Although the proposed grading system is intended for clinical and research purposes and has been available for several years, large cohort studies comparing somatosensory function of clinically diagnosed neuropathic pain patients and patients categorized according to the new grading system are not available. As the neuropathic pain is characterized by both, positive and negative sensory phenomena, it is critical for those phenomena to be captured and, for their optimal utility, to be measured quantitatively. Screening tools for neuropathic pain have been recommended by the NeuPSIG guidelines and include the LANSS and S-LANSS, the NPQ, the DN4, painDETECT and ID-Pain18. Since 10–20% of patients with neuropathic pain will not be detected by these questionnaires it is obvious that these questionnaires cannot replace clinical examination and judgement [5]. Thus, clinical examination is a crucial part of the diagnostic process in neuropathic pain, with sensory testing being the most important factor [5].

The German Research Network on Neuropathic Pain (DNFS) established a standardized Quantitative Sensory Testing (QST) protocol which allows a comprehensive somatosensory characterisation of chronic neuropathic pain patients, using reference values from healthy volunteers [6], [7]. This protocol uses 13 different mechanical and thermal stimuli (e.g. graded von Frey filaments, pin-prick devices, a pressure algometer, and quantitative thermo-testing). It takes about 30 minutes to test one location of the body in healthy volunteers and about 45 minutes in patients. This QST battery tests different sub-modalities of nerve fibres involved in the transduction of sensory information from the periphery to the spinal cord such as Aβ-fibre, Aδ-fibre and C-fibre [6], [7].

There is a long tradition of quantitative measurement of somatic sensory function, well documented in a number of publications [8], [9], [10], [11] and it has been shown to be adequate with respect to reliability and validity [12]. Several publications show that also QST is valid, reliable and sensitive to quantify sensory abnormalities [13], [14], [15], [16].

The ultimate goal of identifying differences in the response to sensory stimuli in neuropathic pain patients is the identification of differences in the mechanisms responsible for generating sensory abnormalities and their subsequent mechanism-based therapy. A recent QST study showed that specific profiles (along thirteen different QST parameters) correspond to the different clinical entities of neuropathic pain [16]. The authors hypothesized that in case of a patient showing many sensory abnormalities, the grading of this patient would fulfil the criteria for ‘probable’ or ‘definite’ neuropathic pain.

Previously, we showed that bilateral somatosensory abnormalities were common in patients with unilateral neuropathic pain [17]. We did not account differences in the numbers of sensory abnormalities at the affected side between the clinical entities of neuropathic pain of our study population. In the present study, we hypothesized that the number of somatosensory abnormalities in patients with clinically diagnosed neuropathy do not differ within the ‘definite’, ‘probable’, ‘possible’ and ‘unlikely’ neuropathic pain grading groups. We also aimed to find QST profile-based corroboration for the new grading system.

We selected a large cohort of patients with clinically confirmed neuropathic pain and subsequently categorized each patient according to the neuropathic pain grading. We examined the painful area using the standardized German Research Network on Neuropathic Pain (DNFS) QST protocol comparing patient values with those obtained from age- and gender-matched healthy volunteers.

## Methods

### Ethics Statement

The study adhered to the declaration of Helsinki and was approved by the independent, medical ethical committee “Stichting Beoordeling Ethiek Bio-Medisch Onderzoek”, P.O. Box 1004, 9400 BA Assen, The Netherlands. This committee is acknowledged by the Central Committee on Research Involving Human Subjects (known by its Dutch initials, CCMO). Patients and healthy controls were recruited from the local region. All participants signed an informed consent form.

### Description of Healthy Controls

Healthy subjects were recruited by advertisement in the local newspaper and were identified according to medical history. Subjects were specifically questioned regarding previous injuries or diseases. The healthy subjects did not use analgesics regularly and were free of medication at the time of the assessments. In total, 209 age- and gender-matched healthy volunteers (age range 20–73 years), of which 138 females (age 45.3±13.4 years) and 71 males (age 48.7±14.0 years) underwent QST assessments on both, the dorsal hand and foot. These body locations have been proposed as reference sites for QST [6]. Since there are no significant differences in QST parameters between the right and left sides of the body in healthy volunteers [6], we obtained QST reference values from one side of the body. In total, 418 QST references from the upper and the lower extremity were obtained.

### Description of the Patient Cohort

Patients with neuropathic pain lasting for more than three months were recruited from the outpatient Department of the Pain Management Unit of the University Medical Center Groningen, The Netherlands. Patients were diagnosed with neuropathic pain by the physicians of the Unit. Neuropathic pain diagnosis was made based on coherent patient history, medical history and physical examination which included neurological function tests. Each clinical diagnosis was additionally confirmed by an experienced pain specialist of the Pain Management Unit based on patient’s files. In total, 84 neuropathic pain patients (age 51.7 year, range 22–75 years), of which 46 females (age 51.4±12.7 years) and 38 males (age 52.0±12.8 years) were assessed. Prior to the QST assessments, patients were asked to rate their ongoing pain level using a Numerical Rating Scale (NRS) of ‘0’ indicating “no pain”, and ‘100’ indicating “most intense pain imaginable”. Patients did not discontinue their regular pain treatment if applicable. Patients underwent the QST assessment, at the area where the most profound pain was experienced (leg: n = 59, arm: n = 25).

### Quantitative Sensory Testing (QST)

The QST battery consisted of seven tests, measuring thirteen parameters and was applied according to the standardized protocol [6]. QST was performed by two research nurses, who underwent a comprehensive training at the DNFS in Germany. All tests were performed at the same research facility of PRA Int., Groningen, The Netherlands. The average room temperature was 23.1°C ±1.7°C.

Thermal QST tests were performed using the Pathway System (Medoc, Israel) and consisted of six parameters: threshold assessments for warm and cold detection (WDT, CDT) and heat pain and cold pain (HPT, CPT). In addition, subjects were asked about paradoxical heat sensations (PHS) during the thermal sensory limen (TSL) procedure of alternating warm and cold stimuli.

Mechanical QST tests consisted of seven different parameters. The mechanical detection threshold (MDT) was determined with modified von Frey filaments (Optihair2-Set, Marstock Nervtest, Germany). The mechanical pain threshold (MPT) was measured with seven weighted pinprick devices (cylindrical, 0.2 mm in diameter flat contact area) with fixed stimulus intensities forces of 8, 16, 32, 64, 128, 256, and 512 mN. Mechanical pain sensitivity (MPS) was assessed using the same pinprick devices to obtain a stimulus–response relation. Dynamic mechanical allodynia (DMA) was assessed as part of the test above, using a set of three light tactile stimulators as dynamic innocuous stimuli: cotton wisp, cotton wool tip fixed to an elastic strip and a standardized brush (SENSElab No. 5, Somedic, Sweden). Vibration detection threshold (VDT) was performed with a Rydel–Seiffer graded tuning fork (64 Hz, 8/8 scale) that was placed over a bony prominence. The wind up ratio (WUR) test was assessed with a pinprick intensity of 256 mN. The pressure pain threshold (PPT) was determined over muscle with a pressure gauge device (FDN200, Wagner Instruments, CT, USA).

**Figure 1:**
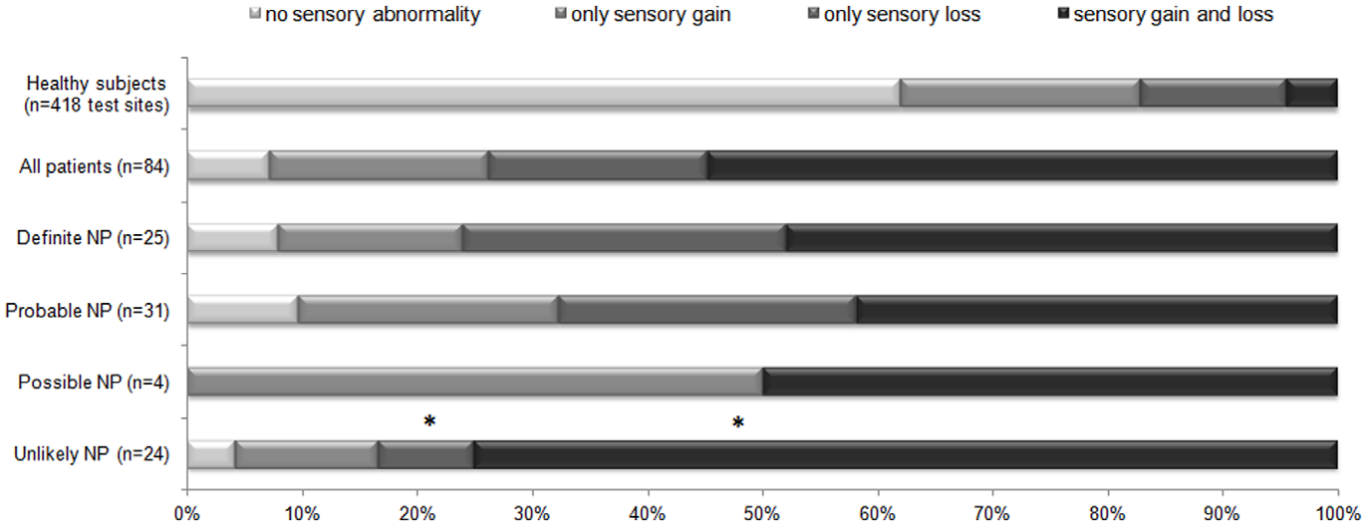
Sensory findings for patients according to neuropathic pain grades and healthy controls. Sensory findings (gain and/or loss of sensory function) in % for healthy controls (n = 209 with 418 test sides), for patients (n = 84) overall and ordered according to their likelihood to be neuropathic pain. “No sensory abnormalities”: none of the Quantitative Sensory Testing (QST) parameters were outside the 95% CI. “Only sensory gain”: at least one QST parameter indicating thermal or mechanical hyperesthesia or hyperalgesia without the presence of hypesthesia or hypoalgesia. “Only sensory loss”: at least one QST parameter indicating thermal or mechanical hypesthesia or hypoalgesia without the presence of hyperesthesia or hyperalgesia. “Sensory gain and loss”: at least one positive sign combined with one negative sign. Wilson estimates of proportions between the groups of definite and probable neuropathic pain and the group of unlikely neuropathic pain for only sensory loss and sensory gain and loss parameter (*p<0.05).

### Neuropathic Pain Grading

For each of the neuropathic pain patients the diagnosis of neuropathic pain was confirmed using the grading system that categorizes neuropathic pain as “definite”, “probable”, “possible” or “unlikely” neuropathic pain [1]. If a patient’s pain complaints did not have a plausible neuroanatomical distribution and lack a history which suggests a relevant lesion or disease, they were regarded as ‘unlikely’ neuropathic. If both requirements were fulfilled the working hypothesis ‘possible’ neuropathic pain was applied. If confirmatory tests such as positive or negative sensory signs confined to the innervations area of the relevant nerve structure and a diagnostic test confirming the lesion or disease were both positive, patients were graded as ‘definite’ neuropathic pain. In case of only one positive confirmatory test, a patient’s pain was graded as ‘probable’ neuropathic. If both confirmatory tests were inconclusive or not performed, a patient’s pain was regarded as unconfirmed and patients were assigned ‘possible’ neuropathic pain. All patients in the present study were allocated to one of the four neuropathic pain grades.

### Z-transformation of QST Data

QST data of patients with neuropathic pain were compared with reference data from gender and age matched healthy volunteers. Since the response to the different QST parameter can be subject to changes over age [6], [7], [16], [18] we divided both, patients and healthy subjects into three age groups each (20–45 years of age, 46–60 years of age and 61–75 years of age). QST values of chronic pain locations on the upper extremities were compared to QST reference values obtained from the dorsal hand of healthy controls (n = 63 for females and n = 29 for males for age group 20–45 years; n = 58 for females and n = 24 for males for age group 46–60 years; n = 17 for females and n = 18 for males for age group 61–75), whereas values from chronic pain locations on lower extremities were compared to reference values obtained from the dorsal foot of healthy controls (n = 63 for females and n = 29 for males for age group 20–45 years; n = 58 for females and n = 24 for males for age group 46–60 years; n = 17 for females and n = 18 for males for age group 61–75). QST values from each patient were transformed to z-scores as described by Rolke et al., 2006 [6]. A score above 1.96 or below −1.96 falls outside the 95% confidence interval of the mean reference value and was considered as a sensory abnormality. Abnormalities were subsequently categorized as either a sensory gain or a sensory loss.

As it never occurs in healthy volunteers that dynamic innocuous stimuli are experiences as painful, the QST parameter “dynamic mechanical allodynia” (DMA) could not be used for z-score analyses. In this case, ratings greater than NRS 10 (scale 0–100) were regarded as clinically relevant and identified as abnormal.

For the QST parameter “Wind-Up Ratio” (WUR), eighteen patients rated the single pinprick stimulus as “0” making ratio calculations (painfulness of one pinprick stimulation vs. painfulness of a train of ten pinprick stimulations) for Wind-Up impossible. For these patients WUR was not used for subsequent analyses. Similar, 31 healthy subjects rated the single pinprick stimulus as “0” making ratio calculations for Wind-up impossible.

### Proportions of Sensory Signs for the Different Neuropathic Pain Grades

Profiles of sensory signs were defined based on the overall sensory numbers and their representation in sensory loss, sensory gain, mixture of sensory loss and gain and no sensory abnormalities. To investigate the differences in the proportions of sensory loss, sensory gain or a mixture of sensory loss and gain for the different neuropathic pain grades, we calculated the 95% confidence intervals of the proportions using the ‘Wilson Estimate’ of proportion [19].

### Correlation between the Number of Sensory Abnormalities and Neuropathic Pain Grades

For each grading, numbers of sensory abnormalities were compared using ANOVA. The total numbers of sensory gain and sensory loss as well as the overall numbers of sensory abnormalities across the thirteen QST parameters were correlated to the different neuropathic pain grades using Spearman correlations.

### Correlation between Background Pain and Neuropathic Pain Grades

For each grading, background pain intensities were compared using ANOVA. To identify a possible relationship between neuropathic pain grade and background pain, Pearson correlation was used. P-values <0.05 were regarded as significant for each statistical test performed.

## Results

### QST Observations in Healthy Controls

From the healthy control cohort (n = 209) investigated in this study, a total of 418 locations were assessed and 5403 measurements were analysed by means of z-score profiling. The total of 1412 measurements for the most affected area were analysed by means of z-score profiling.

### Sensory Abnormalities in Healthy Controls

Although the majority of the QST results obtained in healthy controls confirmed normal sensory function for this cohort, incidental sensory abnormalities (4.3%) were observed for all QST parameters with the exception of DMA. Out of the total of 418 different body areas that were tested across all healthy controls 62% (259 locations) showed normal sensory function and 38% (159 locations) showed a sensory abnormality for at least one QST parameter. Sensory abnormalities were regarded as sensory gain in 21%, sensory loss in 13% and a mixture of sensory gain and sensory loss in 4% of the cases (Fig. 1).

### Demographics of Patients

Demographic data of the patients are shown in Table 1. Apart from two patients, all patients reported ongoing spontaneous pain ranging from 3 to 100 (Mean 63.2±22.2 SD) on a 0–100 NRS just before the QST assessment took place.

**Table 1:** Patient characteristics.

ID	Gender	Age	PainNRS (0–100)	Cause of Pain	ClinicalDiagnose	Grading1	Grading2	Grading3	Grading4	Grading:Conclusion	Numbers ofabnormalities
1	M	62	50	Polyneuropathy	polyneuropathy	yes	yes	yes	positive	definite NP	3
2	F	43	60	Post stroke pain	central pain	yes	yes	yes	positive	definite NP	6
3	M	52	75	Spinocerebellar ataxia	central pain	yes	yes	yes	positive	definite NP	6
4	F	57	80	Diabetic polyneuropathy	polyneuropathy	yes	yes	yes	positive	definite NP	1
5	F	55	90	Herniated nucleus pulposus	peripheral nerve injury	yes	yes	yes	positive	definite NP	2
6	F	53	50	TH12 fracture	spinal cord injury	yes	yes	yes	positive	definite NP	4
7	F	52	80	Sepsis and organ failures	polyneuropathy	yes	yes	yes	positive	definite NP	5
8	F	71	60	Failed back surgery	peripheral nerve injury	yes	yes	yes	positive	definite NP	4
9	F	51	75	Peripheral nerve entrapment	peripheral nerve injury	yes	yes	yes	positive	definite NP	4
10	F	72	50	Failed back surgery	peripheral nerve injury	yes	yes	yes	positive	definite NP	0
11	M	41	60	Failed back surgery	peripheral nerve injury	yes	yes	yes	positive	definite NP	3
12	M	49	40	Accident with trauma	peripheral nerve injury	yes	yes	yes	positive	definite NP	4
13	F	43	80	Postsurgical pain	CRPSII	yes	yes	yes	positive	definite NP	5
14	M	53	70	Polyneuropathy	polyneuropathy	yes	yes	yes	positive	definite NP	6
15	M	36	50	Accident with trauma	peripheral nerve injury	yes	yes	yes	positive	definite NP	5
16	M	52	75	Myelopathy	spinal cord injury	yes	yes	yes	positive	definite NP	3
17	M	46	0	Cruris fracture	peripheral nerve injury	yes	yes	yes	positive	definite NP	4
18	M	66	75	Polyneuropathy	polyneuropathy	yes	yes	yes	positive	definite NP	1
19	M	58	40	Herniated nucleus pulposus	peripheral nerve injury	yes	yes	yes	positive	definite NP	0
20	F	65	70	Herniated nucleus pulposus	peripheral nerve injury	yes	yes	yes	positive	definite NP	4
21	F	42	70	Peripheral nerve entrapment	peripheral nerve injury	yes	yes	yes	positive	definite NP	5
22	M	38	90	Accident with trauma	peripheral nerve injury	yes	yes	yes	positive	definite NP	1
23	F	43	100	Cervical myelopathy	peripheral nerve injury	yes	yes	yes	positive	definite NP	6
24	F	75	80	Herniated nucleus pulposus	peripheral nerve injury	yes	yes	yes	positive	definite NP	3
25	M	46	65	Failed back surgery	peripheral nerve injury	yes	yes	yes	positive	definite NP	2
26	F	37	90	Postsurgical pain	peripheral nerve injury	yes	yes	yes	none	probable NP	4
27	F	48	65	Accident with trauma	peripheral nerve injury	yes	yes	yes	negative	probable NP	0
28	F	46	70	Accident with trauma	peripheral nerve injury	yes	yes	yes	negative	probable NP	1
29	M	56	80	Postsurgical pain	peripheral nerve injury	yes	yes	yes	none	probable NP	5
30	M	55	40	Accident with trauma	peripheral nerve injury	yes	yes	yes	none	probable NP	4
31	F	53	80	Radiotherapy	peripheral nerve vinjury	yes	yes	yes	none	probable NP	3
32	M	26	75	Accident with trauma	peripheral nerve injury	yes	yes	yes	none	probable NP	4
33	F	56	3	Postsurgical pain	peripheral nerve injury	yes	yes	yes	none	probable NP	2
34	F	59	60	Postsurgical pain	peripheral nerve injury	yes	yes	yes	none	probable NP	4
35	F	25	70	Accident with trauma	peripheral nerve injury	yes	yes	yes	none	probable NP	3
36	F	41	70	Postsurgical pain	peripheral nerve injury	yes	yes	yes	negative	probable NP	5
37	F	66	70	Postsurgical pain	peripheral nerve injury	yes	yes	no	positive	probable NP	2
38	M	40	60	Amputation	peripheral nerve injury	yes	yes	yes	negative	probable NP	2
39	F	62	80	Failed back surgery	peripheral nerve injury	yes	yes	yes	none	probable NP	5
40	F	46	85	Postsurgical pain	peripheral nerve injury	yes	yes	yes	negative	probable NP	2
41	F	54	65	Diabetic polyneuropathy	polyneuropathy	yes	yes	yes	none	probable NP	4
42	F	46	40	Amputation	peripheral nerve injury	yes	yes	yes	none	probable NP	3
43	M	63	80	Postsurgical pain	peripheral nerve injury	yes	yes	yes	negative	probable NP	1
44	M	26	85	Accident with trauma	peripheral nerve injury	yes	yes	yes	negative	probable NP	4
45	F	27	70	Femur fracture	peripheral nerve injury	yes	yes	yes	negative	probable NP	8
46	M	62	70	Accident with trauma	peripheral nerve injury	yes	yes	yes	negative	probable NP	1
47	M	58	80	Postsurgical pain	peripheral nerve injury	yes	yes	yes	negative	probable NP	0
48	F	58	90	Postsurgical pain	peripheral nerve injury	yes	yes	yes	none	probable NP	3
49	F	41	70	Metacarpal fracture	peripheral nerve injury	yes	yes	yes	none	probable NP	2
50	M	57	75	Tibia fracture	peripheral nerve injury	yes	yes	yes	none	probable NP	6
51	M	57	40	Polyneuropathy	polyneuropathy	yes	yes	yes	none	probable NP	6
52	M	73	70	Polyneuropathy	polyneuropathy	yes	yes	yes	none	probable NP	0
53	M	24	50	Postsurgical pain	peripheral nerve injury	yes	yes	yes	none	probable NP	9
54	F	61	20	Diabetic polyneuropathy	polyneuropathy	yes	yes	yes	none	probable NP	3
55	F	75	50	Failed back surgery	peripheral nerve injury	yes	yes	yes	negative	probable NP	5
56	F	44	45	Postsurgical pain	peripheral nerve injury	yes	yes	yes	none	probable NP	4
57	M	47	50	Postsurgical pain	peripheral nerve injury	yes	yes	no	none	possible NP	4
58	M	51	70	Postsurgical pain	peripheral nerve injury	yes	yes	no	none	possible NP	6
59	M	59	60	Accident with trauma	peripheral nerve injury	yes	yes	no	negative	possible NP	2
60	F	52	100	Failed back surgery	peripheral nerve injury	yes	yes	no	none	possible NP	5
61	M	43	60	Accident with trauma	peripheral nerve injury	yes	no	yes	negative	unlikely NP	6
62	F	43	75	Meralgia paresthetica	peripheral nerve injury	yes	no	yes	none	unlikely NP	1
63	M	75	65	Postsurgical pain	peripheral nerve injury	no	yes	no	none	unlikely NP	2
64	M	54	75	Postsurgical pain	peripheral nerve injury	no	yes	no	none	unlikely NP	3
65	F	59	75	Ischemic CVA	central pain	yes	no	no	none	unlikely NP	0
66	M	37	60	Accident with trauma	spinal cord injury	no	yes	no	negative	unlikely NP	4
67	F	65	50	Amputation	peripheral nerve injury	no	yes	no	negative	unlikely NP	4
68	M	59	55	Borrelia infection	polyneuropathy	no	yes	no	none	unlikely NP	2
69	F	36	70	Cruris fracture	peripheral nerve injury	no	no	no	none	unlikely NP	8
70	M	51	80	Failed back surgery	peripheral nerve injury	no	no	no	negative	unlikely NP	4
71	F	39	80	Postsurgical pain	peripheral nerve injury	no	no	no	none	unlikely NP	4
72	M	42	70	Failed back surgery	peripheral nerve injury	no	no	no	none	unlikely NP	2
73	F	47	80	Accident with trauma	peripheral nerve injury	no	no	no	none	unlikely NP	2
74	F	66	90	Postsurgical pain	peripheral nerve injury	no	no	no	none	unlikely NP	5
75	M	71	20	Polyneuropathy	polyneuropathy	no	no	no	none	unlikely NP	7
76	F	46	75	Accident with trauma	peripheral nerve injury	no	no	no	none	unlikely NP	3
77	F	22	0	Postsurgical pain	peripheral nerve injury	no	no	no	none	unlikely NP	4
78	F	49	50	Accident with trauma	peripheral nerve injury	no	no	no	negative	unlikely NP	2
79	F	50	10	Radiotherapy	peripheral nerve injury	no	no	no	none	unlikely NP	1
80	F	75	90	Polyneuropathy	polyneuropathy	no	no	no	positive	unlikely NP	7
81	M	73	10	Peripheral nerve entrapment	peripheral nerve injury	no	no	no	negative	unlikely NP	7
82	M	62	80	Polyneuropathy	polyneuropathy	no	no	no	none	unlikely NP	2
83	F	49	30	Failed back surgery	peripheral nerve injury	no	no	no	negative	unlikely NP	4
84	M	57	50	Polyneuropathy	polyneuropathy	no	no	no	negative	unlikely NP	2

Demographic patient overview; Patient ID, gender and age are indicated. Patient’s rating of ongoing pain prior to Quantitative Sensory Testing (QST) using a Numeric Rating scale (NRS) indicating “0” as “no pain” and “100” as the “most intense pain imaginable”. Cause of pain and clinical diagnosis is indicated. For allocating patients pain complaints as neuropathic pain a grading system was applied [1]. This grading determine with a greater level of certainty whether a pain condition is neuropathic. To increase likelihood of neuropathy grading requires that pain in plausible neuroanatomical distribution (Grading 1), that there is a history for a lesion or disease (Grading 2), sensory signs are in a neuroanatomical plausible distribution (Grading 3) and the presence of a positive confirmatory test (Grading 4) (none indicates that no test was performed). Number of abnormalities refers to the number of QST parameter exceeding CI 95% of z-scores at the affected side.

### Clinical Diagnosis of Neuropathic Pain

The aetiology of patient’s pain in our sample was diverse. The largest subgroup developed pain after a surgical intervention (20) followed by patients who had a trauma (16). Other causes of pain were polyneuropathy (12), failed back surgery (10), pain after fracture (6), Herniated Nucleus Pulposus (HNP) (4), spinal cord injury (3), peripheral nerve entrapment (3), central pain (3), amputation (3), Radiotherapy (2), and pain after infection (2). The clinical diagnoses of patients included peripheral nerve injury (63), polyneuropathy (14), spinal cord injury (3), central pain (3) and complex regional pain syndrome (CRPS) (1) (see Table 1).

### Grading of Neuropathic Pain

Patient’s pain was graded into ‘definite neuropathic’ (n = 25), ‘probable neuropathic’ (n = 31), ‘possible neuropathic’ (n = 4) and ‘unlikely neuropathic’ (n = 24) according to the classification by Treede and colleagues [1]. Thus, of the 84 neuropathic pain patients investigated, 67% were graded as having ‘definite’ and ‘probable’ neuropathic pain. Out of this group 45% were accounted as ‘definite’ neuropathic. For patients graded as ‘probable’ neuropathic pain, 65% (n = 20) a diagnostic test was not performed and 35% (n = 11) had a negative outcome of the diagnostic test. Interestingly, in one patient with ‘probable’ neuropathic pain grading the diagnostic test was positive but the confirmatory test was negative (Table 1).

All four patients graded as ‘possible’ neuropathic pain did not have their sensory signs in a neuroanatomical confined territory. For this patient group more confirmatory and diagnostic work would be necessary to advance this group to ‘probable’ neuropathic pain. 30% of the clinically diagnosed neuropathic pain patients were graded as ‘unlikely’ neuropathic pain. This was due to the fact that there was a lack of history of patient’s lesion or disease and their pain complaints were not in a plausible neuroanatomical distribution (Table 1).

### Sensory Abnormalities in Neuropathic Pain

For the 84 patients investigated in this study, 1092 QST data measurements were obtained. In patients with neuropathic pain, sensory abnormalities were observed in all QST parameters. In our patient cohort, 93% had at least one QST abnormality. From these patients 54% had a mixture of sensory gain and loss, 20% had only sensory gain (hyperalgesia) and 19% had only sensory loss (hypesthesia) (Fig. 1).

### Proportions of Sensory Signs for the Different Neuropathic Pain Grades

The profiles of sensory signs in ‘definite’ and ‘probable’ neuropathic pain were not significantly different, but different from the ‘unlikely’ grade. This latter difference was due to an increase of a mixture of sensory gain and loss and a decrease in frequency of sensory loss only for the ‘unlikely’ grade compared to the ‘definite’ and ‘probable’ neuropathic pain grade (all p<0.05) (Fig. 1).

These results indicate that profiles of sensory signs for ‘definite’ and ‘probable’ neuropathic pain differ from the profiles for the ‘unlikely’ grade.

### Individual QST Parameters

**Figure 2:**
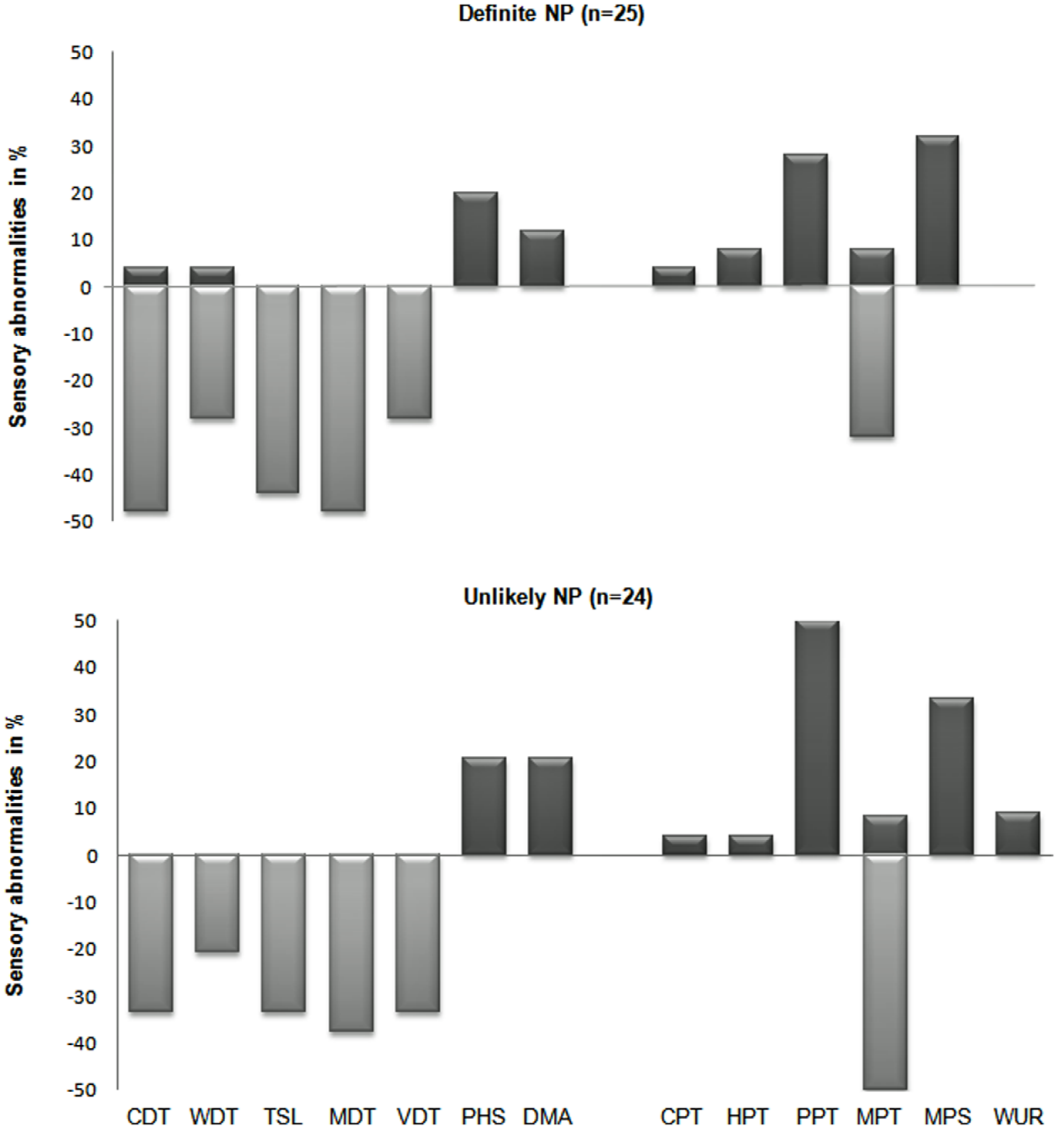
QST z-score abnormalities for patients graded as ‘definite’ and ‘unlikely’ neuropathic pain. Quantitative Sensory Testing (QST) z-score abnormalities in % for ‘definite’ neuropathic pain (top) and ‘unlikely’ neuropathic pain (bottom) grades. QST parameter are ordered as sensory parameters: Cold Detection Threshold (CDT), Warm Detection Threshold (WDT), Thermal Sensory Limen (TSL), Mechanical Detection Threshold (MDT), Vibration Disappearance Threshold (VDT), Paradoxical Heat Sensation (PHS), Dynamic Mechanical Allodynia (DMA) and nociceptive parameters: Cold Pain Threshold (CPT), Heat Pain Threshold (HPT), Pressure Pain Threshold (PPT), Mechanical Pain Threshold (MPT), Mechanical Pain Sensitivity (MPS) and Wind Up Ratio (WUR). Z-scores with positive sensory signs (gain of sensory function) plotted upwards and negative sensory signs (loss of sensory function) plotted downwards. Absence of DMA is normal and therefore no negative sign possible.

For the different grading groups of neuropathic pain similar patterns of the distribution of sensory abnormalities were observed. Since the different neuropathic pain grades showed comparable profile only the two most distant opponents i.e. ‘definite’ and ‘unlikely’ neuropathic pain, are displayed for illustration in Figure 2. All neuropathic pain grades showed predominantly sensory gain changes for nociceptive QST parameters (CPT, HPT, PPT, MPS, WUR) reflecting hyperalgesia, whereas the non-nociceptive parameters (CDT, WDT, TSL, MDT, VDT) reflected hypesthesia (Fig. 2). For the nociceptive parameters CPT and HPT, thermal pain thresholds were decreased indicating thermal hyperalgesia. An increased pain due to blunt pressure (PPT) and an increased sensitivity to mechanical pain (MPS) were observed indicating only hyperalgesia for these parameters. For MPT a greater incidence for mechanical hypo- than hypersensitivity was detected. WUR was always increased and not decreased indicating hypersensitivity. For every patient sensory loss was only observed for the non-nociceptive CDT indicating a thermal hypesthesia. In addition, thermal hypesthesias were observed in most of the patients for WDT and TSL. For MDT predominantly a sensory loss was observed indicating a mechanical hypesthesia. It was not possible to detect hyperesthesia for VDT as the maximal value of 8/8 measured by the tuning fork was within the normal range. PHS and DMA were found to be increased within all grading of neuropathic pain.

Our results show that sensory abnormalities for the individual QST parameters are remarkably similar between the grades of neuropathic pain. These similarities were also reflected in the distribution for nociceptive and non-nociceptive QST parameters for the different neuropathic pain grades.

### Number of Sensory Abnormalities in Relation to Neuropathic Pain Grading

The number of sensory abnormalities in neuropathic pain patients varied between 0 and 9 for the thirteen QST parameters (see Table 1). In three out of the four grading categories, i.e. ‘unlikely’, ‘definite’ and ‘probable’ neuropathic pain, a small fraction of patients did not show any sensory abnormality upon undergoing the complete QST monitoring.

**Figure 3:**
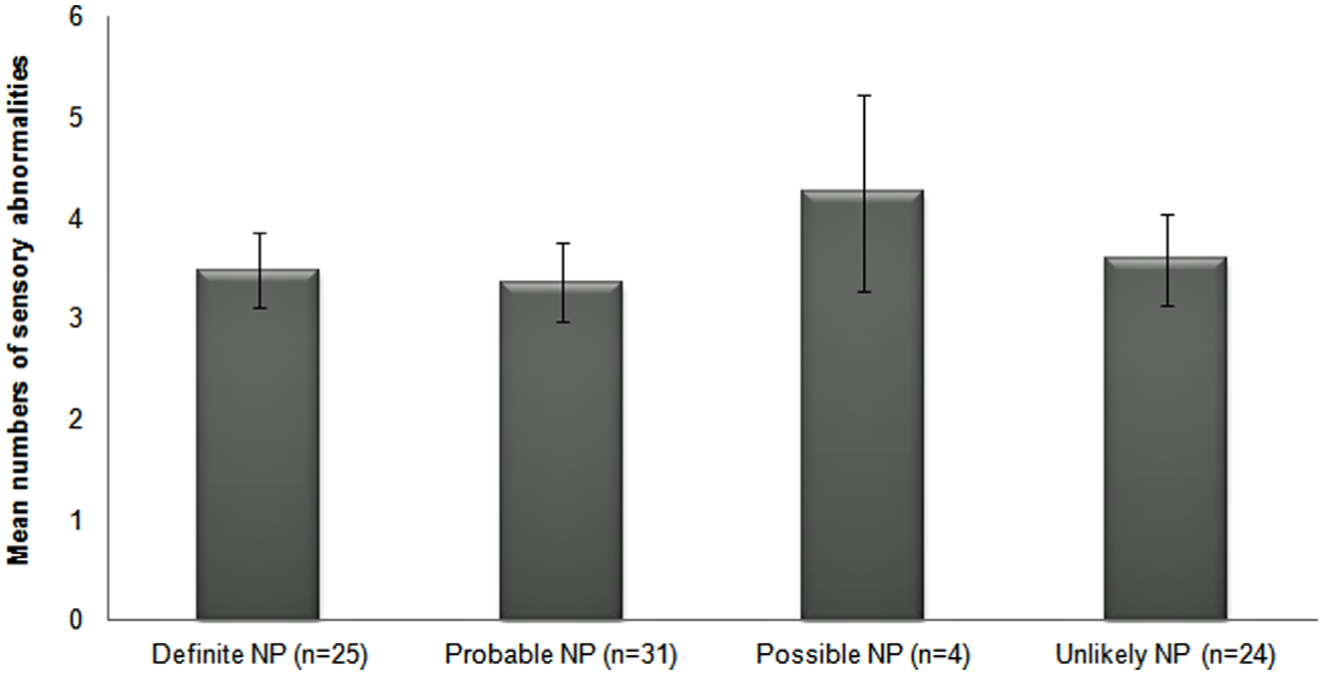
Numbers of sensory abnormalities for patients according to neuropathic pain grades. Numbers of sensory abnormalities (sensory gain and loss) for patients (n = 84) graded as ‘definite’, ‘probable’, ‘possible’ and ‘unlikely’ neuropathic pain; Mean values ± SEM.

When comparing the number of sensory abnormalities in the different categories of graded patients, the mean number of abnormalities for the group of patients graded as ‘definite’ neuropathic pain was 3.5 (SD ±1.9). Similar numbers were also found for the group of patients with ‘probable’ and ‘unlikely’ neuropathic pain, 3.4 (SD ±2.2) and 3.6 (SD ±2.1) respectively. Slightly higher was the number of abnormalities observed for the group of patients graded as ‘possible’ neuropathic pain (4.3, SD ±1.7). This increase is not significant compared to patient groups graded as ‘definite’, ‘probable’ or ‘unlikely’ neuropathic pain using ANOVA (Fig. 3). The total numbers of sensory gain and sensory loss as well as the overall numbers of sensory abnormalities across the thirteen QST parameters were not correlated to the different neuropathic pain grades using Spearman correlations.

### Background Pain in Relation to Neuropathic Pain Grading

Except for two patients, all patients reported ongoing spontaneous pain (NRS mean 63.3, SD±22.2) before the QST assessment took place (see Table 1). For patients graded as ‘definite’ neuropathic pain the mean NRS score for spontaneous pain were 65.4 (SD±20.2). Slightly higher pain levels were reported by patients graded as ‘possible’ neuropathic pain (70.0, SD±21.6). Patient’s graded as ‘probable’ and ‘unlikely’ reported slightly lower pain levels of 64.5 (SD±20.2) and 58.3 (SD±26.3), respectively. However, these differences were not significant (ANOVA). Pearson correlation revealed no significant difference between neuropathic pain grade and background pain.

## Discussion

Investigating the somatosensory profiles of patients using QST showed that somatosensory abnormalities are a common feature in neuropathic pain. Applying the grading system for neuropathic pain revealed similar numbers of somatosensory abnormalities across the four different grading categories. Analysing the profile of sensory signs showed that overall the ‘definite’ and ‘probable’ neuropathic pain groups have a similar profile. The categories of a mixture of sensory gain and loss as well as sensory loss only differed significantly for these groups compared to the ‘unlikely’ grade. There was no significant correlation between background pain and the different neuropathic pain grades.

The grading system allows a separation of neuropathic and non-neuropathic pain based on profiles but not on the total amount of sensory abnormalities. Thus, the suggestion that patient selection based on grading of neuropathic pain may provide a more homogenous group of neuropathic pain patients for research and for clinical studies is only partly supported by the findings of this study.

### Clinical Diagnoses and Grading of Neuropathic Pain

All patients investigated in the present study were diagnosed with neuropathic pain based on clinical presentation. Neuropathic pain is notoriously difficult to diagnose and due to the lack of a specific diagnostic tool a grading system to categorize pain as ‘definite’, ‘probable’, ‘possible’ and ‘unlikely’ neuropathic was proposed [1]. For 71% of the patients investigated, a direct history of a relevant lesion or disease and plausibly distributed pain was confirmed. These patients were subsequently graded at least as ‘possible’ neuropathic pain. In 23% of the patients such a plausible distribution of their pains was not identified, therefore these patients were graded as ‘unlikely’. For 25% of the patients no direct history of a relevant lesion or disease was identified. For these patients a greater degree of certainty than ‘unlikely’ could not be reached. Overall, 29% of patients gained the neuropathic pain grade ‘unlikely’.

A small group of patients (5%) were graded as ‘possible’ neuropathic pain. Here confirmatory tests were either negative or had not yet been performed, therefore this group of patients is regarded as having ‘unconfirmed’ neuropathic pain. This status is difficult to judge since clinical investigations determine this category. Any additional positive confirmatory test could change the status to neuropathic pain e.g. ‘probable’ and/or ‘definite’. On the other hand, it has not been described how to proceed with the grading if future confirmatory tests were negative. For that reason the comparison of ‘definite’ and ‘probable’ versus ‘unlikely’ neuropathic pain grading is the most valuable for this paper.

According to the classification criteria only definite and probable grades are to be regarded as neuropathic pain [1]. Therefore, 33% of patients investigated should be regarded as having non-neuropathic pain. Apparently, there is a mismatch in the outcome ‘neuropathic pain’ between the clinical observations/diagnosis and the grading system. Reason for such differences could lie in the fact that the grading system relies on a direct relationship between cause of pain and its neuroanatomical plausible distribution to exclude ‘unlikely’ neuropathic pain grade. It could be argued that the grading system is “biased” towards precisely defined neuropathic pain entities. Once a distinct clinical entity is confirmed an increase in the certainty of neuropathic pain is almost an “epiphenomenon” since confirmatory evidence is often part of the assessment. Examples include neuropathic pain after a known surgical nerve lesion or postherpetic neuralgia after shingles. From a clinical perspective such a direct relationship is sometimes difficult to establish. For example, a large group in the present study are postsurgical pain patients (n = 20) which were diagnosed clinically with peripheral nerve injury. Out of this pool, thirteen patients were graded as ‘definite’ and ‘probable’, two patients as ‘possible’ and five patients as ‘unlikely’ neuropathic pain. For grading purposes, it has been suggested that the distribution of pain or hyperalgesia does not necessarily need to be identical to the innervations area of a peripheral nerve or root, but it should be in a distribution that is typical for the underlying disorder [1]. This is easy to recognise in well-defined diseases such as postherpetic neuralgia where central sensitization might influence the distribution of sensory abnormalities. In contrast it is less clear in patients with postsurgical pain since it has been not established if damage to tissues other than nerves causes neuropathic pain after surgery [20].

Overall, 67% of the patients were graded as ‘definite’ and ‘probable’ neuropathic pain. Interestingly, different clinical neuropathic pain entities were found consistently within the different grades of neuropathic pain.

### Somatosensory Function in Healthy Controls

Z-score transformation of QST data revealed one or more somatosensory abnormalities in 38% of the healthy control group. This number is in line with previous findings of 41% abnormalities using the QST protocol [16].

For healthy volunteers, abnormalities were observed across all QST parameters with the exception of DMA. The detected sensory abnormalities reflected gain of function for the most part, some loss of function and in a minority a mixture of gain and loss of function (Fig. 2).

### Somatosensory Function in Neuropathic Pain Patients

As expected, the large majority (93%) of neuropathic pain patients showed sensory abnormalities. Previously, a similar percentage (92%) of patients with at least one QST abnormality were reported [16]. Given the fact that for 7% of the patients, no abnormality could be detected, QST and the cut-off of 95% CI of the mean reference values might be more stringent than clinical examination.

In accordance with previous studies, sensory loss was predominantly found in non-nociceptive parameters [16], [21], which could be associated with central or peripheral neuronal damage leading to ongoing pain via increased ectopic activity [22], [23], [24]. Sensory gain was predominantly found in nociceptive parameters which could be associated with peripheral sensitization and/or altered central processing [10], [25], [26], [27], [28]. Overall, there was good agreement between our estimates of the expected range of sensory abnormalities in the general neuropathic pain patient population and those reported by Maier [16].

### Somatosensory Function Across the Grading of Neuropathic Pain

The pattern of sensory abnormalities for nociceptive and non-nociceptive parameters did not differ for the different neuropathic pain grades. A similar distribution of nociceptive and non-nociceptive parameters was previously reported in neuropathic pain patients [16].

Recently, Maier and colleagues reported in a QST study of 1236 neuropathic pain patients that profiles of sensory abnormalities differ in the neuropathic pain conditions [16]. Differences in profiles of sensory abnormalities were also observed in our study based on the grading system of neuropathic pain. The presence of sensory gain and loss and only sensory loss was similar for the grade of ‘definite’ and ‘probable’ but was significantly different to the grade ‘unlikely’ neuropathic pain.

Our results indicate that the grading system allows a separation of neuropathic and non-neuropathic pain based on profiles of sensory abnormalities.

### Background Pain, Number of Sensory Abnormalities and Neuropathic Pain Grading

QST revealed that the numbers of sensory abnormalities did not differ between the different neuropathic pain grades. This observation challenges the hypothesis that the number of sensory abnormalities is positively related to neuropathic pain grades using the grading system [16].

In a study with 618 neuropathic- and non-neuropathic pain patients, Dworkin and colleagues showed that pain intensity, unpleasantness, quality, and spatial characteristics differed significantly between these groups [29]. In the present study we have assessed the intensity of background pain prior to QST. There was no correlation between background pain intensity and numbers of somatosensory abnormalities in patients clinically diagnosed as neuropathic pain or for the different grades of neuropathy.

Sensory testing in healthy subjects and patients using a reaction time-sensitive “method of limits” procedure reported poor reliability [30]. Therefore, its results are highly dependent on the subjects’ motor abilities and attention [8]. In addition, the majority of patients investigated (91%) used their regular medication when the QST assessment took place which could have even more influenced the assessments of WDT, CDT, HPT and CPT and subsequently the sensory profiles detected. This is not ideal, but it reflects the most common situation in which QST testing is performed, clinically. Furthermore, all categories of the neuropathic pain grading include patients with medication. Apart from the ethical aspect of drug withdrawal leading to increased pain, many neuropathic pain medications have long elimination times and possible active metabolites, making drug withdrawal prior to testing both unwarranted and unpractical.

In conclusion, our results indicate that there is a mismatch between clinical neuropathic pain diagnoses and neuropathic pain grading outcome. Only 60% of patients with clinically diagnosed neuropathy were categorized as ‘definite’ and ‘probable’ neuropathic pain patients. Even if such a stringent grading system may provide advantages in selecting homogenous groups for clinical research, numbers of somatosensory abnormalities within the different certainties of neuropathic pain are remarkably similar. The only significant finding to differentiate “true” neuropathic pain from “unlikely” neuropathic pain was the difference in somatosensory profiles, in particular with regard to the presence of the mixture of sensory gain and loss and only sensory loss. Neuropathic pain grades as well as numbers of sensory abnormalities were not correlated with patients reported background pain intensity.
